# Cementless, modular, distally fixed stem in hip revision arthroplasty: a single-center study of 132 consecutive hips

**DOI:** 10.1007/s00590-017-2013-x

**Published:** 2017-07-12

**Authors:** Ali Hashem, Ammar Al-Azzawi, Hasan Riyadh, Sebastian Mukka, Arkan Sayed-Noor

**Affiliations:** Department of Surgical and Perioperative Sciences, Umeå Univeristy, Umeå, Sweden

**Keywords:** Hip revision, Cementless stem, Distally fixed, Outcome, Complication, Survival

## Abstract

**Purpose:**

The use of cementless, modular, distally fixed stem in hip revision arthroplasty has increased during the last decades. We aimed to analyze the early and late postoperative complications, re-operation rate, and survival rate of the MP stem operated at our county hospital with relatively limited caseload.

**Methods:**

In this retrospective study, we included 132 hips operated with MP stem between January 2007–2014. An independent observer reviewed patients’ medical records in July 2015 (18–102 months postoperatively, median 52.5) to collect the following data: age, sex, American Society of Anesthesiologists (ASA) class, body mass index, indication of revision, type of operation, early and late complications, re-operation rate, and mortality during study period.

**Results:**

The commonest indication for MP stem operation was aseptic loosening (72%). We found early and late postoperative complications in 29% of cases. The most common complication was prosthetic dislocation (8%), followed by intra-operative peri-prosthetic fracture (5%). The commonest indication for MP re-operation was soft tissue revision for infection (7%) followed by closed reduction for prosthetic dislocation (6%). We found no correlation between the age, sex, ASA class, and type of operation and the re-operation risk. Only one prosthesis was extracted giving a survival rate for 99% for the study period.

**Conclusion:**

This study showed good results of the MP prosthesis with reasonable complication and re-operation rates and negligible extraction rate, indicating the good performance of this implant even when used in the setting of a county hospital with limited caseload.

## Introduction

Primary total hip arthroplasty (THA) is a successful and cost-effective procedure. As life expectancy increases and patients receiving THA live longer, the need for revision hip arthroplasty is expected to increase steadily during the coming decades [1, 2]. In revision hip surgery, the reconstruction challenge posed to the orthopedic surgeon is high since revision patients can suffer general debility, compromised soft tissue envelope, muscular deficiency and cavitary defects in the proximal femoral shaft, as well as cortical perforations. The technical options available for addressing this problem are divided between cemented and cementless. Long cemented stems to cover the proximal femur defect can be used on its own or over impaction grafting of morcellized allograft [3]. Cementless options include fully coated monoblock stems or modular distally fixed femoral stems. The modularity of the latter option allows better restoration of the leg length, femoral offset, Collum-center diaphysis (CCD) angle and anteversion. Wagner in 1987 was the first to describe a cementless revision stem with anchoring the stem in the intact femur distally [4]. The advantage of such a distally fixed femoral stems is that strong fixation distal to the bone defect allows for regeneration of bone in the proximal femur. In recent years, stems based on the principles of Wagner have been introduced and developed. The Link MP reconstruction hip stem is one of those implants offering the option of adjusting the length of the head-neck segment after the stem has been securely implanted distally. A few reports, mostly based on register data or university hospital production, have documented its utility in clinical practice. The purpose of this study was to analyze the early and late postoperative complications, re-operation rate, and survival rate of MP stem operated at our county hospital with relatively limited caseload.

## Patients and methods

The study was conducted according to the Declaration of Helsinki, and the local ethics committee approved the protocol.

### Patients

Our hospital, Sundsvall Hospital, is a county hospital with a catchment area of approximately 160.000 inhabitants. Four general orthopedic surgeons with interest in hip surgery do approximately 25–40 hip revisions annually. In this study, patients between January 2007–2014 with hip revision receiving an MP stem were included. All patients received the same peri-operative antibiotics and thrombosis prophylaxis. Antibiotic prophylaxis was given in three doses of 2 g of Cloxacillin (Ekvacillin^®^; Meda, Sweden) at 0.5 h before and 1.5 and 9.5 h after the start of surgery. Klindamycin (Dalacin^®^, Pfizer AB, Sweden) was used in patients with anaphylaxis to penicillin. Thrombo-prophylaxis with subcutaneous high molecular weight heparin (5000 IE of Fragmin^®^ Pfizer AB, Sweden) was given for 10 days. Postoperative physiotherapy included anti-dislocation movement restriction and weight bearing as tolerated with crutches or a walker for support was recommended for the first 6–12 weeks.

In July 2015 (18–102 months postoperatively, median 52.5), an independent observer (a senior resident) who was not involved in the management of any patient reviewed the prospectively documented patients’ medical records to collect the following data: age, sex, American Society of Anesthesiologists (ASA) class, body mass index (BMI), operated side, indication of revision, type of operation (stem revision or stem and cup revision), early and late complications, re-operation rate, and mortality during study period.

### Implant

**Fig. 1:**
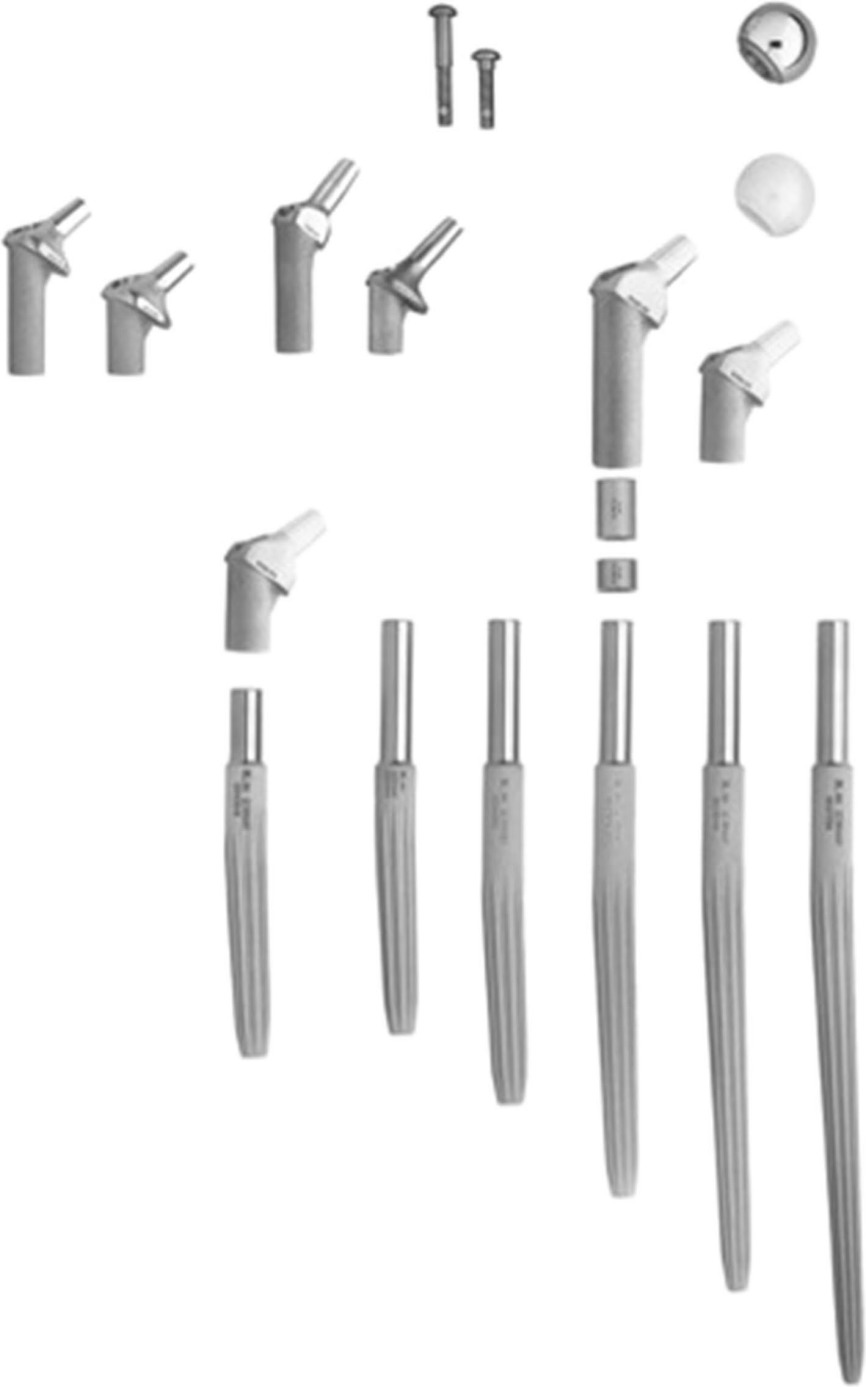
Available components of the MP stem

The implant used in these operations was the Link MP reconstruction prosthesis (Waldemar LINK, Hamburg, Germany, Fig. 1). It consists of a long femoral component made of titanium alloy with a grit blast finish giving it a microporous structure of 70-mm pore size. This enhances non-cement anchorage within the distal femur. The implant is composed of four main parts: a head, a proximal head-neck segment, optional intermediate spacer rings, and a long cementless stem distally. The stem can be implanted in the position which best conforms the anatomy and proximal modular segments optimize the configuration of the prosthesis. Stem length can be determined during preoperative planning and varies between 210, 250, 290, and 330 mm, while stem diameter ranges between 14, 16, 18, 20, 22.5, and 25 mm. The stem carries an angle of 3° proximally mimicking the natural anterior bow of the femur so that introduction of the prosthesis can be regularly achieved without an osteotomy. Distally the stem is tapered and has longitudinal ribs that provide rotational stability.

Once the stem is implanted firmly, the spacer rings can afford an added length by 10, 20, and 30 mm. The proximal modular head-neck segment is then added which is available in CCD angles of 126° and 135°. The base of this segment is toothed so it allows the desired anteversion angle to be “dialed in.” The neck has a taper of 12/14 mm onto which fits a modular head. The choice available in head components exists in cobalt–chromium alloy or aluminum oxide ceramic, in head-neck lengths between 46, 50, 56, and 60 mm. In the present study, a head diameter of 32 mm was used in all patients.

### Statistics

The collected data were presented as a mean value and standard deviation. The survival of the MP stem over the study period was measured with a Kaplan–Meier Survival Probability Estimate. A regression analysis was used to check for any correlation between the age, sex, ASA class and type of operation and the re-operation risk. A *p* value <0.05 was considered significant.

## Results

We included 127 patients (132 hips; 5 patients received bilateral prostheses) in the study. The demographic data of the included patients are shown in Table 1.

**Table 1:** Study population characteristics

Age (years)	70 (40–89)
Sex	
Male	78 (59%)
Female	54 (41%)
Side	
Right	62 (49%)
Left	58 (46%)
Bilateral	6 (5%)
ASA class	
1–2	95 (73%)
3–5	36 (27%)
BMI	28 (19.6–46.6)

Continuous variables are presented as means and range

Sixty-one hips (46%) were operated with cup and MP stem revision and 71 hips (54%) only with MP stem revision. All patients were operated on using the postero-lateral approach. The extended trochanteric osteotomy was used in five hips (3.5%). The indications for revision are included in Table 2.

**Table 2:** List of indications for MP stem use

Aseptic loosening	95	72%
Septic loosening	15	11.5%
Failed treatment of hip fracture	12	9%
Peri-prosthetic fracture around a primary THA	7	5.5%
Primary THA stem fracture	3	2%

We found early and late postoperative complications in 29% of cases. The most common complication was prosthetic dislocation (8%). The list of complications is shown in Table 3.

**Table 3:** List of early and late postoperative complications

No complication	94	71%
Dislocation	11	8%
Intra-operative peri-prosthetic fracture	7	5%
Deep infection	6	4%
Superficial infection	9	7%
Chronic prosthetic infection	2	1.5%
Trochanteric bursitis	2	1.5%
Drop foot	1	1%

At least one re-operation was conducted in 22% of the cases; the most common type of re-operation was soft tissue debridement due to superficial and deep infection. The indications for re-operation are shown in Table 4.

**Table 4:** List of re-operation for the MP stems

No re-operation	103	78%
Soft tissue revision for infection	9	7%
Closed reduction for prosthetic dislocation	8	6%
Plate fixation for fracture	6	5%
Open reduction for prosthetic dislocation	1	0.8%
Prosthetic revision	1	0.8%
Lengthening of ilio-tibial band for trochanteric bursitis	2	1.5%
Cup revision due to instability	1	0.8%

Only 1 MP prosthesis (0.8%) was extracted due to deep infection that could not be treated with soft tissue debridement and antibiotics. During the study period, 26 patients died (5 of them died within 18 months postoperatively). This resulted in a survival rate of 84% (95% CI 79–90). To study the possible influence of age, sex, ASA class, and type of operation (stem revision vs. cup and stem revision) on the re-operation risk, a regression analysis was conducted (Table 5). No such influence was found (*p* > 0.05).

**Table 5:** Logistic regression analysis showed no influence of the included parameters on the re-operation risk (*p* > 0.05)

	Coff.	95% CI	*p* value
Age	1.0	0.94–1.02	0.4
Sex			
Male	1.0	–	–
Women	0.8	0.36–2.0	0.7
ASA class			
1–2	1.0	–	–
3–5	1.9	0.81–4.79	0.13
Type of revision			
Stem	1.0	–	–
Stem and cup	0.8	0.34–1.83	0.58

## Discussion

This retrospective study showed very good results of the MP prosthesis with reasonable complication and re-operation rates and negligible extraction rate. The commonest indication for MP prosthesis was aseptic loosening, the commonest complication was instability and the commonest re-operation type was soft tissue debridement due to peri-prosthetic infection. These results resemble previous studies [5–7] and indicate the good performance of this implant even when used in the setting of a county hospital with limited caseload.

The philosophy of MP prosthesis relies on prosthetic bony fixation distal to the isthmus of the proximal femoral diaphysis. A minimal of 4-cm press-fit fixation between the stem and diaphysis is required to achieve axial and rotational stability. By this way, surgeons can use this prosthesis even in the presence of bony defects in the proximal femur. Osteo-integration in the proximal femur with some bony growth at the site of defects can occur. In the present study, 70% of MP prostheses were implanted in patients with aseptic loosening of cemented stem. In these patients, extraction of old cement gives bony defects and weakness and therefore compromises the possibility of using conventional stems. The same problem can be encountered in patients with septic loosening and fractures of the proximal femur with distortion of the calcar.

The fluted distally fixed stems can subside in the femoral canal a few millimeters during the first weeks–months postoperatively, if the initial press-fit fixation is inadequate. This can compromise the long-term survival [6, 8, 9]. In the present study, we did not include the postoperative radiographs to evaluate this parameter or because of the lack of uniform routine for timing and standardization of the radiographic evaluation in our patients. Another important aspect is the risk for intra-operative peri-prosthetic fractures. This happens when the pressure applied on the diaphysis is increased by reaming or prosthetic insertion. In many cases, surgeons choose to do prophylactic wiring to give additional stability to the diaphysis. In this study, we had six intra-operative peri-prosthetic fractures. The treatment of these fractures is usually technically demanding with high rate of mechanical failure and prosthetic subsidence. Therefore, every effort should be made to prevent this complication. Another complication is instability. Indeed, this complication is one of the commonest complications in many reports [5–7, 10] including the present one. The soft tissue insufficiency, mainly defective gluteal muscles, and inadequate restoration of soft tissue tension are predisposing factors to instability. Preoperative planning is essential to choose the right stem position and version and stem neck length and angle. When instability takes place, a detailed workup is necessary to choose the right treatment. The modularity of the stem allows changing the version, angle, and length of the stem neck to improve the prosthetic stability. Other methods may involve cup revision to dual-mobility cup or cup with constrained liner. The latter implies coupling of the head of the prosthesis to the cup to prevent the head from dislocating from the cup. This coupling is associated with high mechanical loading, which can give rise to cup dislodgment. We have limited experience with this type of coupling. Another drastic complication is peri-prosthetic infection. Many patients are elderly and with co-morbidities (36% of patients had ASA class 3–5). Furthermore, local factors such as excessive fibrous tissue and soft tissue and bony defects increase the risk of superficial and deep infection. Early infections should be treated with aggressive debridement and antibiotics [11, 12]. When the infection is more than 4–8 weeks, a biofilm is formed. This provides a suitable media for the bacteria where antibiotics cannot work. Fortunately, we had only one patient who sustained such an infection and who needed prosthetic extraction and resection arthroplasty. We succeeded to treat our infected prostheses with debridement and antibiotics. Measures such as prophylactic peri-operative antibiotics, careful surgical technique, and postoperative wound care are minimal requirements in this regard.

The overall survival of MP prosthesis in this study was 99%, while the re-operation rate was 22%. These very good results concur with those reported by others. Weiss et al. [5] showed a 98% survival of the MP prosthesis at 5-year and 10% re-operation rate. Implant failure (modular junction) was no problem in our series, which is in accordance with other studies. Tamvakopoulos et al. [13] reported comparable findings with an overall survival of 92.5% of the MP system at an average follow-up of 5.6 years in 40 cases. Kwong et al. [8] evaluated 143 patients at an average of 3.3 years with 97% component survival using the same stem. Wirtz et al. [14] reported on the results of 142 hip revisions using the distally fixated MRP-Titan stem. The survival was 95% at an average of 2.3 years of follow-up.

Previous studies have reported pertinent information related to the results of the present study. Rodriguez et al., for instance, observed 97 hips for an average of 45 months, with clinical improvement of Harris hip score from 36 to 84 (range 54–99). Radiographically, 93 hips were considered stable, with no circumferential lucencies at the distal fixation surface. Three hips migrated and required revision, along with one peri-prosthetic fracture. Five other hips had nonprogressive migration of 1–2 mm. Also, McIinns reported on 70 MP stems with a mean follow-up of 47 months. Combined metaphyseal/diaphyseal bone loss was present preoperatively in 36 (51%) of 70 hips. Three hips (4.3%) were re-revised or in need of re-revision, and worst-case survival was 87%. The mean postoperative patient-assessed Oxford Hip Score was 21.1. Restoration of proximal bone was noted in 56%. Complications included mean subsidence of 9.9 mm, dislocation in 7 (10%) of 70 hips, and fracture or cortical perforation in 17 (24.2%) of 70. Furthermore, Amanatullah et al. used MP prosthesis in 92 patients with a mean clinical follow-up of 6.4 years. A total of 47 patients had peri-operative complications, 27 of which required further surgery. Most of these further operations involved retention of a well-fixed femoral stem, and 88/92 femoral components (97%) remained in situ. Of the four components requiring revision, three were revised for infection and were well fixed at the time of revision; only one (1%) was revised for aseptic loosening. The most common complications were postoperative instability (17 hips, 19%) and intra-operative femoral fracture during insertion of the stem (11 hips, 12%). Diaphyseal stress shielding was noted in 20 hips (22%). At the final follow-up 78% of patients had minimal or no pain. The mean HHS, which was available for 88 patients, at final follow-up was 69 points. There were 26 with good or excellent HHSs (30%), 24 with fair (27%), and 38 with poor scores (43%).

The present study has a few limitations owing to its retrospective design. The lack of functional and radiological measures makes the evaluation of study outcome results incomplete. We chose not to include these parameters because of insufficiency of data documentation in some patient records. The strength of this study is the relatively large sample size and the independency of the observer who collected the data. The authors think that the results of the present study reflect the actual everyday practice and results more than registry data, which can be jeopardized by missing data and underreporting.

In conclusion, our results indicate a very good performance of the MP prosthesis with comparable survival and complication rates compared with other reports. This motivates us to continue using this implant for our patients, even with if our caseload is relatively low. Further prospective studies are warranted to address the clinical and radiological outcome.
